# Glymphatic clearance estimated using diffusion tensor imaging along perivascular spaces is reduced after traumatic brain injury and correlates with plasma neurofilament light, a biomarker of injury severity

**DOI:** 10.1093/braincomms/fcad134

**Published:** 2023-04-25

**Authors:** Tracy Butler, Liangdong Zhou, Ilker Ozsahin, Xiuyuan Hugh Wang, Jacob Garetti, Henrik Zetterberg, Kaj Blennow, Keith Jamison, Mony J de Leon, Yi Li, Amy Kuceyeski, Sudhin A Shah

**Affiliations:** Brain Health Imaging Institute, Department of Radiology, Weill Cornell Medicine, New York NY 10044, USA; Brain Health Imaging Institute, Department of Radiology, Weill Cornell Medicine, New York NY 10044, USA; Brain Health Imaging Institute, Department of Radiology, Weill Cornell Medicine, New York NY 10044, USA; Brain Health Imaging Institute, Department of Radiology, Weill Cornell Medicine, New York NY 10044, USA; Brain Health Imaging Institute, Department of Radiology, Weill Cornell Medicine, New York NY 10044, USA; Department of Psychiatry and Neurochemistry, Institute of Neuroscience and Physiology, The Sahlgrenska Academy at the University of Gothenburg, Mölndal 40530, Sweden; Clinical Neurochemistry Laboratory, Sahlgrenska University Hospital, Mölndal 41345, Sweden; Department of Neurodegenerative Disease, UCL Institute of Neurology, Queen Square, London WC1E 6BT, UK; UK Dementia Research Institute at UCL, London W1T 7NF, UK; Hong Kong Center for Neurodegenerative Diseases, Clear Water Bay, Hong Kong 999077, China; Wisconsin Alzheimer's Disease Research Center, University of Wisconsin School of Medicine and Public Health, University of Wisconsin-Madison, Madison, WI 53726, USA; Department of Psychiatry and Neurochemistry, Institute of Neuroscience and Physiology, The Sahlgrenska Academy at the University of Gothenburg, Mölndal 40530, Sweden; Clinical Neurochemistry Laboratory, Sahlgrenska University Hospital, Mölndal 41345, Sweden; Brain Health Imaging Institute, Department of Radiology, Weill Cornell Medicine, New York NY 10044, USA; Brain Health Imaging Institute, Department of Radiology, Weill Cornell Medicine, New York NY 10044, USA; Brain Health Imaging Institute, Department of Radiology, Weill Cornell Medicine, New York NY 10044, USA; Brain Health Imaging Institute, Department of Radiology, Weill Cornell Medicine, New York NY 10044, USA; Brain Health Imaging Institute, Department of Radiology, Weill Cornell Medicine, New York NY 10044, USA

**Keywords:** traumatic brain injury, glymphatic, perivascular, clearance, MRI

## Abstract

The glymphatic system is a perivascular fluid clearance system, most active during sleep, considered important for clearing the brain of waste products and toxins. Glymphatic failure is hypothesized to underlie brain protein deposition in neurodegenerative disorders like Alzheimer’s disease. Preclinical evidence suggests that a functioning glymphatic system is also essential for recovery from traumatic brain injury, which involves release of debris and toxic proteins that need to be cleared from the brain. In a cross-sectional observational study, we estimated glymphatic clearance using diffusion tensor imaging along perivascular spaces, an MRI-derived measure of water diffusivity surrounding veins in the periventricular region, in 13 non-injured controls and 37 subjects who had experienced traumatic brain injury ∼5 months previously. We additionally measured the volume of the perivascular space using *T*_2_-weighted MRI. We measured plasma concentrations of neurofilament light chain, a biomarker of injury severity, in a subset of subjects. Diffusion tensor imaging along perivascular spaces index was modestly though significantly lower in subjects with traumatic brain injury compared with controls when covarying for age. Diffusion tensor imaging along perivascular spaces index was significantly, negatively correlated with blood levels of neurofilament light chain. Perivascular space volume did not differ in subjects with traumatic brain injury as compared with controls and did not correlate with blood levels of neurofilament light chain, suggesting it may be a less sensitive measure for injury-related perivascular clearance changes. Glymphatic impairment after traumatic brain injury could be due to mechanisms such as mislocalization of glymphatic water channels, inflammation, proteinopathy and/or sleep disruption. Diffusion tensor imaging along perivascular spaces is a promising method for estimating glymphatic clearance, though additional work is needed to confirm results and assess associations with outcome. Understanding changes in glymphatic functioning following traumatic brain injury could inform novel therapies to improve short-term recovery and reduce later risk of neurodegeneration.

## Teaser

Using an MRI technique called diffusion tensor imaging along perivascular space to estimate brain glymphatic clearance, Butler *et al.* demonstrate clearance reductions in subjects with traumatic brain injury as compared with healthy controls and in association with a blood biomarker of injury severity.

## Graphical Abstract

**Graphical Abstract:**
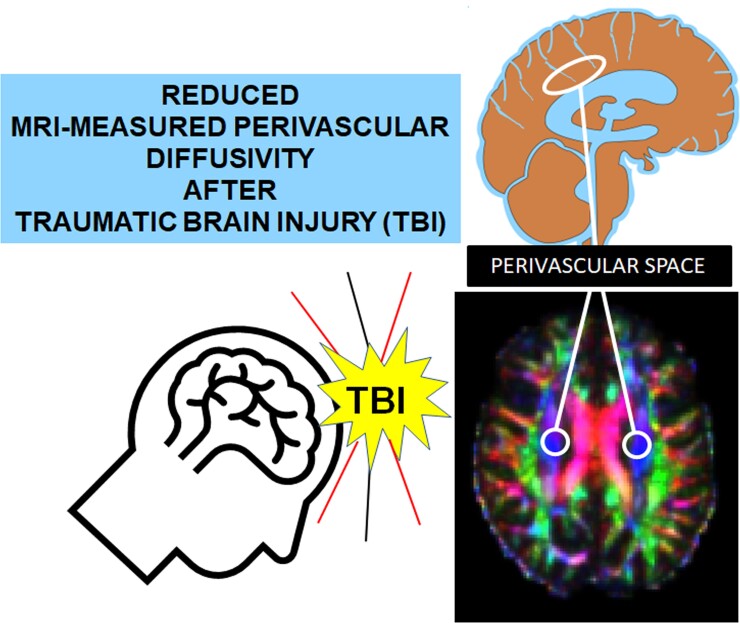

## Introduction

Brain clearance can be broadly defined as the removal of waste from the brain via multiple, overlapping systems including active and passive transport at brain barriers, diffusion and the glymphatic system.^1–4^ The glymphatic system involves subarachnoid cerebrospinal fluid flowing into the brain alongside arteries, mixing with brain interstitial fluid (ISF) containing waste products and then flowing out of the brain along veins. This convective perivascular flow allows more rapid waste clearance than would be possible with diffusion alone. Impaired clearance has been hypothesized to be part of the pathophysiology underlying the buildup of the toxic proteins amyloid-β (Aβ) and tau causing Alzheimer’s disease^1,4^

Clearance may also be important after traumatic brain injury (TBI), when there is neuronal debris and release of proteins including Aβ and tau which need to be cleared. TBI-induced clearance deficits may be one explanation for TBI increasing later risk for Alzheimer’s disease and other dementias.^5,6^ In an animal model, glymphatic clearance disruption promoted tau deposition and impaired cognitive recovery.^7^ Understanding the role of clearance after TBI could provide novel therapeutic targets to enhance TBI recovery and reduce future risk of neurodegeneration.

**Figure 1:**
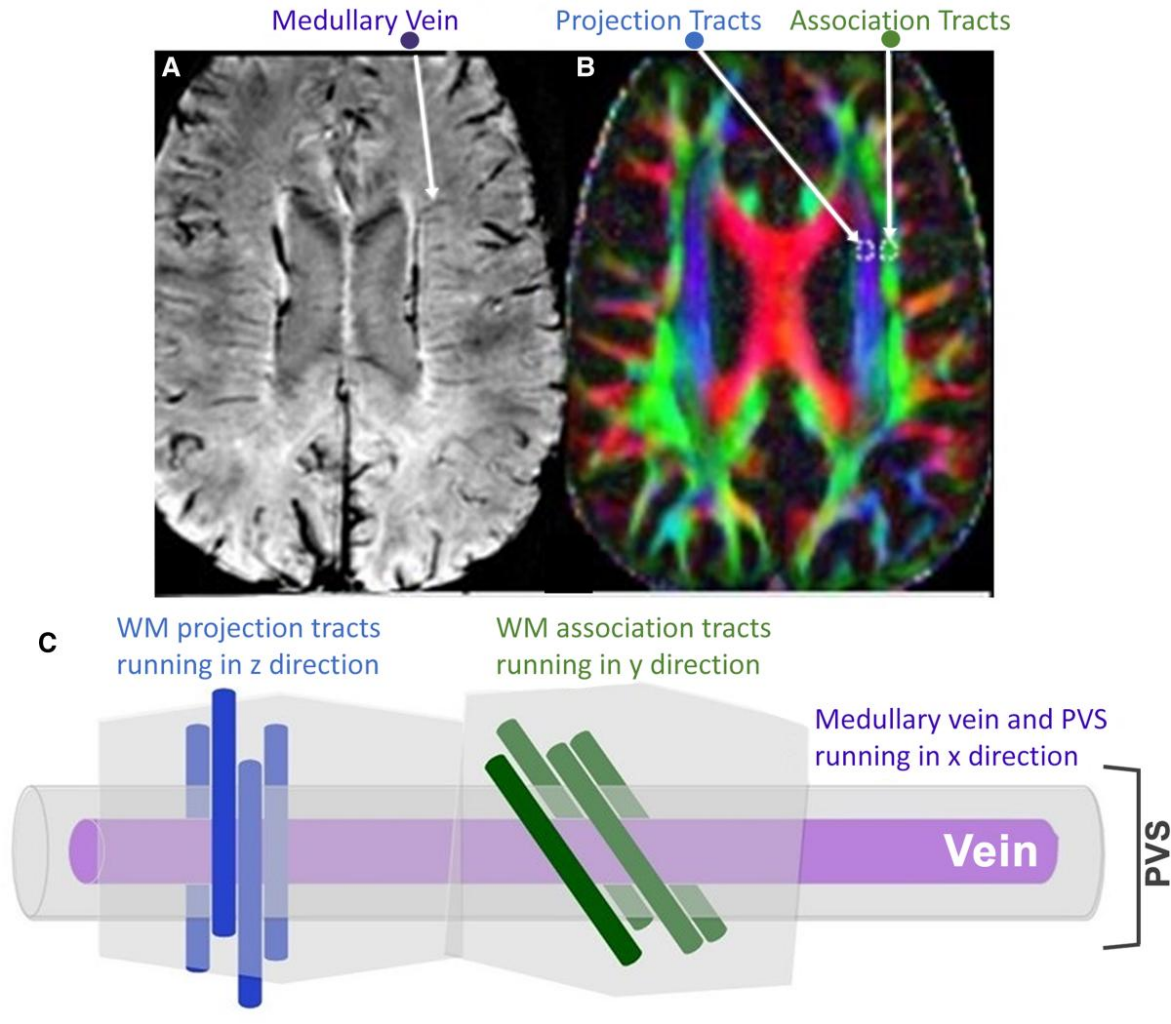
**DTI-ALPS: diffusion tensor imaging along perivascular spaces. (A)** Susceptibility weighted image showing clear medullary veins. **(B)** ROI placement within fibre tracts on colour-coded FA maps at the medullary veins. **(C)** A schematic adapted from Taoka *et al*.^13^ of the perivascular space running parallel to the medullary vein and therefore orthogonal to the principal fibre tract orientations. WM means brain white matter.

Three prior studies have shown enlarged perivascular spaces (PVS)—the location where cerebrospinal fluid mixes with ISF to facilitate clearance of waste—in TBI.^8–10^ This is considered evidence of possible glymphatic stasis and failed clearance.^11^ However, a static measure of PVS does not reflect the dynamic process of fluid flow. A new technique called diffusion tensor imaging along perivascular spaces (DTI-ALPS) quantifies actual water diffusion with PVS.^12,13^ Specifically, DTI-ALPS allows evaluation of diffusivity parallel to PVS surrounding medullary veins at the level of the lateral ventricles. As shown in Fig. 1, because the direction of PVS is orthogonal to projection and association white matter fibre tracks, axial diffusion at high *b*-value (to suppress intravascular venous flow) is uniquely sensitive to perivenous flow.^12,13^ Decreased glymphatic function was very recently demonstrated using this method in subjects with Alzheimer’s disease^14^ and TBI.^15^ Here, we apply DTI-ALPS to assessing glymphatic function in subacute TBI and in relation to blood levels of neurofilament light protein (NfL)—a robust biomarker of TBI severity.^16,17^ We also measured PVS volume. We hypothesized that glymphatic functioning estimating using DTI-ALPS would be reduced in subjects with TBI as compared with healthy controls.

## Materials and methods

TBI participants were recruited through inpatient rehabilitation units and trauma departments between 2018 and 2022. Control subjects were recruited through local advertisements. All subjects provided informed consent prior to participation, and all study activities were approved by Weill Cornell Medicine’s Institutional Review Board. TBI subjects had sustained a complicated mild [Glasgow Coma Scale (GCS) score of 13–15 with evidence of intracranial lesion on acute neuroimaging] or moderate–severe TBI (GCS ≤ 12) within the prior 6 months. They were scanned ∼5 months after injury. Controls were free from medical and psychiatric illness and substance abuse.

### MRI data acquisition

Anatomical *T*_1_-weighted MPRAGE (0.8-mm isotropic) and multi-shell diffusion-weighted MRI (1.5-mm isotropic, *b* = 1500, 3000 and 98 directions per shell) was acquired with a multiband acceleration factor 4 on a 3T Siemens Prisma scanner with a 32-channel head coil. Diffusion data were collected with both anterior–posterior and posterior–anterior phase-encoding, with TE/TR = 89.2/3230 ms. *T*_2_-weighted image was acquired using T2SPACE sequence with matrix size 208 × 300 × 320 and isotropic 0.8-mm voxel size for PVS segmentation.

### DTI-ALPS processing

Diffusion MRI (dMRI) data were corrected for susceptibility-induced geometric and eddy current distortions and intervolume subject motion using the top-up and eddy toolboxes.^18^ The preprocessed dMRI data were used to fit diffusion tensors and obtain fractional anisotropy (FA) and mean diffusivity (MD) maps for each subject in the directions of the *x*- (right–left, $D_{xx}$), *y*- (anterior–posterior, $D_{yy}$) and *z*-axes (inferior–superior, $D_{zz}$). $D_{xx}\;$ corresponds to the direction of vessels in the periventricular white matter. Considering that the perivascular glymphatic system runs along these vessels, $D_{xx}\;$ is assumed to reflect water diffusivity along the glymphatic system.

As shown in Fig. 1, each participant’s FA map was colour coded in RGB style using the first eigenvector and 5-mm-diameter square regions of interest (ROIs) were manually placed bilaterally in the projection area (predominantly in *z*-axis direction) and association areas (predominantly in *y*-axis direction) at the level of the lateral ventricle. Average FA and MD in each ROI were recorded as measures of white matter integrity. The diffusivity values along the *x*-, *y*- and *z*-axes within the ROIs were obtained for each participant. The ALPS index was calculated as a ratio of the mean of the *x*-axis diffusivity in the projection area ($D_{xx,proj}$) and *x*-axis diffusivity in the association area ($D_{xx,assoc}$) to the mean of the *y*-axis diffusivity in the projection area ($D_{yy,proj}$) and the *z*-axis diffusivity in the association area ($D_{zz,assoc}$).^12,13^ No laterality difference were observed, so left and right ALPS indices were averaged for data analysis. A higher ALPS index indicates greater diffusivity along PVS, while an index close to 1.0 reflects minimal diffusion.

### Measurement of PVS volume and count

We measured whole brain-‘enhanced’ PVS volume using an automated image processing method that increase the contrast ratio between PVS and surrounding tissue by combining *T*_2_- and *T*_1_-weighted MRI.^19^ We also manually counted the number of PVS on a single *T*_2_-weighted axial slice in the centrum semiovale one slice above the superior extent of the lateral ventricle and calculated a commonly-used PVS severity score (0–4) based on this count.^20^

### NfL measurement

Plasma NfL concentration in blood drawn at the time of MR scanning was measured using single-molecule array (Simoa; Quanterix; Billerica, MA, USA) performed at the Clinical Neurochemistry Laboratory, University of Gothenburg, Sweden, in a single analytical run, following established methods.^16^

### Statistical analyses

Analyses were performed in SPSS version 27; *t*-test and chi-square were used to assess group demographic differences.

Pearson test was used to assess correlations between injury features (GCS, time between TBI and assessment) and ALPS index, PVS measures and NfL.

Analysis of covariance (ANCOVA) was used to compare the ALPS index, PVS measures (volume, count and score), measures of white matter integrity (Fa and Md) and plasma NfL between TBI subjects and controls while controlling for subject age.

Multiple regression was used to assess the contribution of ALPS index, PVS volume and age to the dependent variable of plasma NfL in subjects with TBI.

## Results

### Subject demographics

Subject information is shown in Table 1. TBI subjects were non-significantly younger than controls (*P* = 0.098). Males were over-represented in the TBI group as compared with controls, though this was not statistically significant (chi-square = 2.183, *P* = 0.14.)

**Table 1:** Participant demographics

	Subjects with TBI	Controls
Sex	28 males (75.7%); *n* = 37	7 males (53.8%); *n* = 13
Age	51.5 (range: 19–85, SD = 16.7); *n* = 37	58.5 (range: 33–86, SD = 15.1); *n* = 13
Time in days since injury	148.7 (range: 62–241, SD = 39); *n* = 37	N/A
Glasgow Coma Scale	12.3 (range: 5–15, SD = 2.9); *n* = 17	N/A

### Correlation between PVS diffusivity (ALPS index) and measures of PVS volume

As expected, PVS volume was highly correlated with PVS count (*R* = 0.901, *P* < 0.001) and score (*R* = 0.842, *P* < 0.001). ALPS Index did not correlate with PVS volume (*R* = −0.108, *P* = 0.46) nor the other PVS measures.

### Effects of injury factors on ALPS index, PVS and NfL

There was an expected trend for lower NfL in association with greater time since injury (*R* = −0.479; *P* = 0.052.) No other correlations (between GCS and ALPS, GCS and NfL, ALPS and time since injury) approached significance (*P* > 0.1).

### Group differences in ALPS index, PVS measures and NfL and age effects

**Figure 2:**
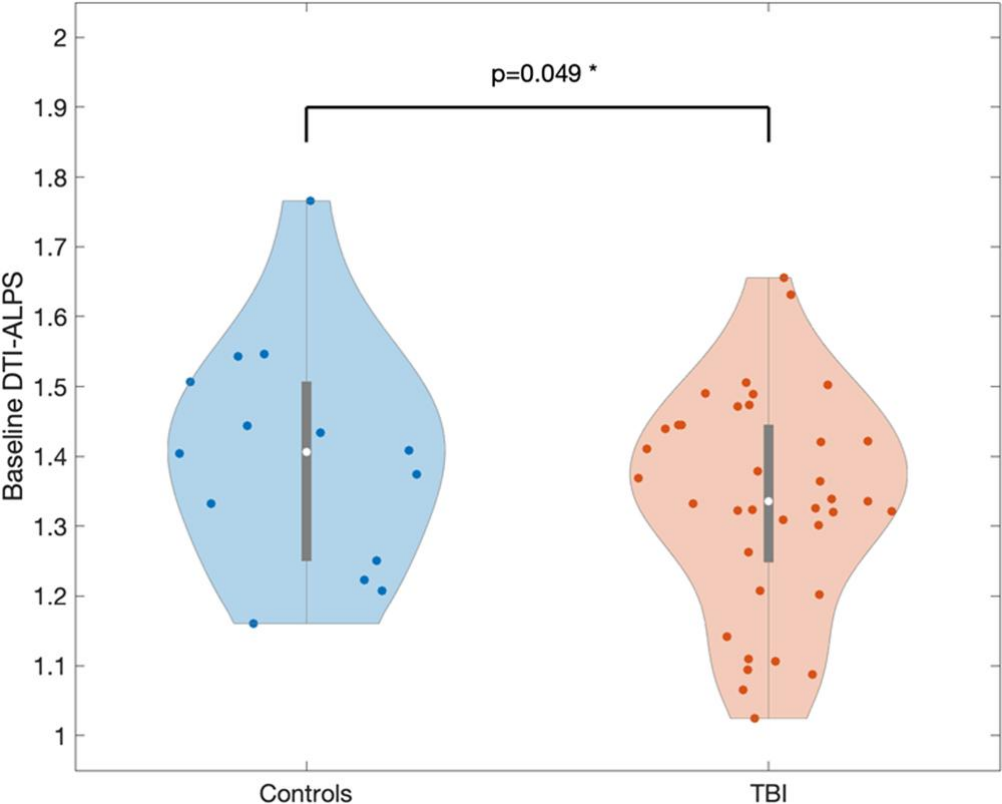
**Violin plot showing distribution of ALPS index in TBI subjects versus controls.** When controlling for age, TBI subjects had significantly lower ALPS index as compared with controls [*F*(1,47) = 4.083, *P* = 0.049], consistent with glymphatic dysfunction.

As shown in Fig. 2, ALPS index was significantly lower in subjects with TBI (*n* = 37) compared with controls (*n* = 13) after controlling for a significant effect of age: [TBI mean = 1.336, SD = 0.155; control = 1.389, SD = 164; *F*(1,47) group effect = 4.083, *P* = 0.049; age effect = 19.349, *P* < 0.001].

There were no group differences between TBI subjects and controls in FA [*F*(1,47) = 2.52, *P* = 0.12] or MD [*F*(1,47) = 1.86, *P* = 0.18] though there was a significant age effect for FA [*F*(1,47) = 19.05, *P* = 0.004].

PVS volume did not differ between TBI subjects and controls: [TBI (*n* = 37) mean = 0.95, SD = 0.77; control (*n* = 13) = 1.10, SD = 1.05; *F*(1,47) group effect = 0.075, *P* = 0.79; age effect = 2.0, *P* = 0.16]. Results were similar when including total intracranial volume as an additional covariate and when replacing PVS volume with PVS count or score.

When controlling for a significant effect of age, subjects with TBI had numerically higher plasma NfL concentration than controls (TBI mean = 90.59, SD = 115.18, *n* = 23; control mean = 22.65, SD = 20.99, *n* = 9), but this group difference was not significant: [F(1,29) NfL = 1.28, *P* = 0.267; age effect =9.936, *P* = 0.004].

### Contribution of ALPS index, PVS volume and age to plasma NfL concentration

**Figure 3:**
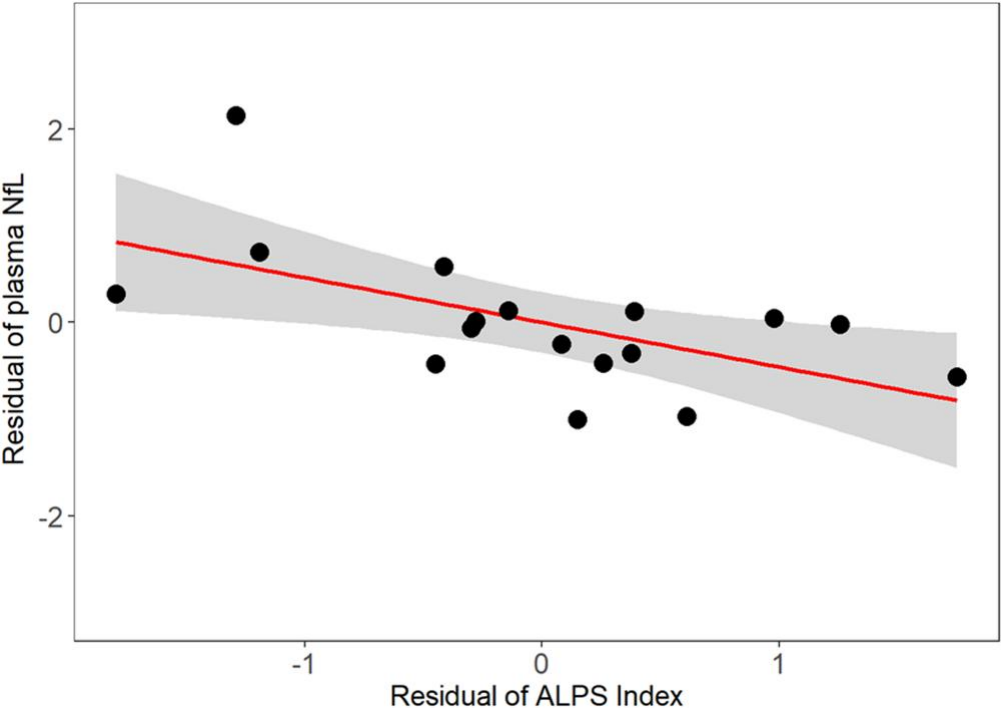
**Partial regression plot of ALPS index with plasma NfL in 17 TBI subjects from multiple regression model also including age and PVS volume.** Lower glymphatic function as estimated by ALPS index is associated with higher NfL, indicative of greater neural injury (ALPS index *b* = −0.482, *P* = 0.039).

In subjects with TBI, a significant regression model (*R*^2^=.607, *F* = 5.665, *P* = 0.014) showed that ALPS index (*b* = −0.482, *P* = 0.039) and age (*b* = −0.773, *P* = 0.003) predicted NfL, but PVS volume did not (*b* = −0.083, *P* = 0.694). Results were similar when using PVS count and score instead of PVS volume. As shown in Fig. 3, lower ALPS index (worse glymphatic function) was associated with higher NfL.

## Discussion

ALPS index,^12,13^ a measure of perivascular diffusivity considered an indicator of the activity of the glymphatic system, was significantly lower in subacute TBI subjects compared with controls, even after controlling for effect of age. Results are in accord with one animal study^7^ and one recent clinical study in a similar population of subjects scanned ∼1 month after TBI.^15^ The correlation of ALPS index with plasma NfL—a biomarker of brain injury severity^16,17^—indicates that greater neuronal damage is associated with worse glymphatic function.

We did not detect a difference between TBI subjects and controls in PVS volume. This contrasts with prior studies showing enlarged PVS in TBI subjects, considered to reflect glymphatic stasis.^8–10^ This discrepancy may be due to our small sample size. The fact that there was a significant difference between TBI subjects and controls in ALPS index—but not PVS—suggests that ALPS may be a more sensitive measure of TBI-related clearance deficits. Similarly, ALPS but not PVS correlated with plasma NfL, considered one of the most sensitive markers of neural damage in TBI.

Mechanisms for decreased clearance after TBI may include sleep disruption, mislocalization of aquaporin-4 water channels involved in glymphatic clearance, protein deposition and sleep disruption.^7^ Impaired clearance after TBI—when cellular debris, blood, inflammatory cells and proteins including the pathognomonic Alzheimer’s disease proteins Aβ and tau need to be cleared—would be expected to affect short-term recovery after injury and contribute to later risk of proteinopathy and neurodegeneration.^21^ Additional study of post-TBI clearance in humans and animal models is warranted to clarify relevant mechanisms and guide targeted therapy. Because sleep is required for optimal clearance,^3^ and TBI is well known to impair sleep,^22^ focusing on improving sleep after TBI may be a promising strategy.^9^

We interpret the negative correlation between ALPS and NfL as reflective of TBI severity, with lower ALPS and higher NfL both indicating greater neural damage. However, these two measures may be related. The movement of brain proteins used as TBI biomarkers from brain to blood depends upon clearance mechanisms including glymphatic function and blood–brain barrier breech to a variable degree. This introduces a potential confound, with blood biomarker levels reflecting both degree of injury and movement from brain to blood, which can be altered by that injury. In an animal model of TBI, impaired clearance has been shown to decrease blood levels of the TBI biomarkers S100β, glial fibrillary acidic protein (GFAP) and neuron-specific enolase to control levels.^23^ However, NfL levels are not considered to be affected by alterations in the blood brain barrier,^24^ and current results suggest that if glymphatic dysfunction causes decreased blood levels of NfL, the effect may be minor. Additional studies including larger numbers of subjects and additional blood and neuroimaging biomarkers reflecting different aspects of clearance are greatly needed. Not accounting for clearance has been proposed as the answer to the question, ‘Why have we not yet developed a simple blood test for TBI?’^23^

ALPS index did not correlate with PVS volume. This is not unexpected as they are very different measures: PVS, visible on *T*_2_-weighted MRI, quantifies the amount of cerebrospinal fluid surrounding small arteries, either automatically over the whole brain (PVS volume) or in the centrum semiovale via manual counting (count and index.) ALPS index, on the other hand, corresponds to water diffusivity around medullary veins within a small, bilateral periventricular region. While a relation between the volume of PVS and diffusivity within it might be expected, such a relation might not be straightforward; while ALPS index is a clear reflection of perivascular diffusivity in one region, with lower diffusivity reflecting pathology, interpretation of PVS volume must account for the apparent contradiction that PVS expansion is considered to facilitate effective perivascular clearance during sleep in animal models,^3^ yet static PVS enlargement in human is considered pathologic and to reflect glymphatic stasis.^11^ Additional work is needed to determine the relation between PVS diffusivity and volume and clarify possible differential relevance of these measure to perivascular clearance after TBI.

The ALPS index was affected by ageing as well as TBI, with lower ALPS index in older subjects. There were also significant age effects on PVS volume and plasma NfL. Clearance is known to decline with ageing.^1,4^ Demonstration of significant group differences and correlation with NfL, after controlling for age, support DTI-ALPS as a sensitive marker of TBI-induced glymphatic deficit.

This study has several limitations. We use DTI-ALPS to measure perivascular glymphatic clearance. However, there is significant controversy about details of fluid clearance, and it remains challenging to assess in both animal and humans.^1,25^ DTI-ALPS measures perivenous diffusivity only in a small periventricular region, and it is implausible to think that glymphatic clearance would be uniform across the brain; whole-brain methods for assessing perivascular diffusivity are needed. Further, DTI-ALPS is considered sensitive only to perivenous diffusivity, not to periarterial clearance, which is considered the main egress route for brain waste by some groups, though this is controversial.^1,26^ The ALPS depends upon the orthogonal orientation of the PVS to white matter tracts and could be affected by axonal damage. Mitigating this possibility, all images were visually inspected for possible white matter damage when ROIs for ALPS calculation were placed, and dMRI measures of axonal integrity (FA and MD) did not differ significantly between TBI subjects and controls.

Other methods for interrogating fluid clearance include PET measurements of radiotracer efflux from ventricles,^27,28,29^ functional MRI measurement of coordinated neural and fluid pulsations linked to and driving cerebrospinal fluid pulsations^30,31^ and dynamic contrast MRI.^32^ Determining which method or combination of methods best reflects clearance in humans is hampered by the absence of a ‘gold-standard’ clearance measure. However, DTI-ALPS has significant face validity as a measure of *actual* diffusivity of fluid along PVS and has been shown to correlate with the rate at which intrathecally injected contrast appears in PVS,^33^ considered one of the most direct measures of perivascular clearance obtainable in humans.^34^

The TBI group was over 75% male, reflecting the epidemiology of moderate TBI.^35^ Larger studies including greater numbers of women are needed to assess possible sex differences in glymphatic function, which may be relevant to important sex differences in TBI mechanisms and outcomes.^36^

The ALPS index difference between TBI subjects and controls was moderate; we hypothesize that this may relate to the timing of the MRI scan: ∼5 months after injury. It is likely that scanning subjects in the acute phase of recovery will capture greater glymphatic impairment that resolves over time. Longitudinal analyses of clearance function changes after TBI are needed.

## Data Availability

The data are available for appropriate request under the agreement of data sharing from the authors' institute.

## Abbreviations

Aβ =: amyloid-β
ANCOVA =: analysis of covariance
dMRI =: diffusion MRI
DTI-ALPS =: diffusion tensor imaging along perivascular spaces
FA =: fractional anisotropy
GCS =: Glasgow Coma Scale
GFAP =: glial fibrillary acidic protein
ISF =: interstitial fluid
MD =: mean diffusivity
NfL =: neurofilament light protein
PVS =: perivascular spaces
ROI =: regions of interest
SD =: standard deviation
TBI =: traumatic brain injury
WM =: brain white matter
